# Longitudinal imaging in *C9orf72* mutation carriers: Relationship to phenotype

**DOI:** 10.1016/j.nicl.2016.10.014

**Published:** 2016-10-22

**Authors:** Mary Kay Floeter, Devin Bageac, Laura E. Danielian, Laura E. Braun, Bryan J. Traynor, Justin Y. Kwan

**Affiliations:** a Motor Neuron Disorders Unit, OCD, NINDS, NIH 10 Center Drive Room 7-5680 Bethesda, MD 20892-1404, United States; b Neuromuscular Disease Research Section LNG, NIA, NIH 35 Convent Drive Room 1A213 Bethesda, MD 20892-3707, United States; c Department of Neurology, University of Maryland, 110 S. Paca Street, Baltimore, MD 21201, United States

**Keywords:** ALS, amyotrophic lateral sclerosis, ALSFRS-R, ALS functional rating scale — revised, ANCOVA, analysis of covariance, ANOVA, analysis of variance, bvFTD, behavioral variant frontotemporal dementia, C9 +,  subjects with *C9orf72* expansion mutations, CSF, cerebrospinal fluid, DRS-2, Mattis dementia rating scale, DTI, diffusion tensor imaging, FBI, frontobehavioral inventory, FDR, false discovery rate correction, FTD, frontotemporal dementia, MRI, magnetic resonance imaging, sALS, sporadic ALS, SD, standard deviation, TIV, total intracranial volume, *C9orf72*, Amyotrophic lateral sclerosis, Frontotemporal dementia, Diffusion tensor imaging, Cortical thickness, Ventricular volume

## Abstract

Expansion mutations in the *C9orf72* gene may cause amyotrophic lateral sclerosis (ALS), frontotemporal dementia (FTD), or mixtures of the two clinical phenotypes. Different imaging findings have been described for *C9orf72*-associated diseases in comparison with sporadic patients with the same phenotypes, but it is uncertain whether different phenotypes have a common genotype-associated imaging signature. To address this question, 27 unrelated *C9orf72* expansion mutation carriers (C9 +) with varied phenotypes, 28 age-matched healthy controls and 22 patients with sporadic ALS (sALS) underwent 3T MRI scanning and clinical phenotyping. Measures of brain volumes and cortical thickness were extracted from T1 images. Compared to healthy controls and sALS patients, symptomatic C9 + subjects had greater ventricular volume loss and thalamic atrophy for age, with diffuse, patchy cortical thinning. Asymptomatic carriers did not differ from controls. C9 + ALS and ALS-FTD patients had less thinning of the motor cortex than sALS patients, but more thinning in extramotor regions, particularly in frontal and temporal lobes. C9 + ALS patients differed from sporadic ALS patients in the thickness of the superior frontal gyrus and lateral orbitofrontal cortex. Thickness of the precentral gyrus was weakly correlated with the revised ALS functional rating scale. Thickness of many cortical regions, including several frontal and temporal regions, was moderately correlated with letter fluency scores. Letter fluency scores were weakly correlated with ventricular and thalamic volume. To better understand how imaging findings are related to disease progression, nineteen C9 + subjects and 23 healthy controls were scanned approximately 6 months later. Ventricular volume increased in C9 + patients with FTD and ALS-FTD phenotypes and remained stable in asymptomatic C9 + subjects. We conclude that diffuse atrophy is a common underlying feature of disease associated with *C9orf72* mutations across its clinical phenotypes. Ventricular enlargement can be measured over a 6-month time frame, and appears to be faster in patients with cognitive impairment.

## Highlights

• Patchy cortical thinning and diffuse atrophy are a hallmark of symptomatic ALS and FTD *C9orf72* mutation carriers.
• Symptomatic *C9orf72* carriers have more atrophy and diffuse thinning than sporadic ALS patients and healthy controls.
• Ventricular enlargement can be detected over a 6-month interval in symptomatic *C9orf72* carriers.
• Impaired letter fluency is associated with diffuse cortical thinning.
• Changes in letter fluency, but not ALSFRS-R are correlated with 6-month ventricular enlargement.

## 1 Introduction

Expansion mutations in the *C9orf72* gene are one of the most common causes of familial amyotrophic lateral sclerosis (ALS) and familial frontotemporal dementia (FTD) in US and European populations (Renton et al., 2011, DeJesus-Hernandez et al., 2011, Majounie et al., 2012). Patients with the *C9orf72* mutation can present with a clinical phenotype of FTD, ALS, or a combination of ALS and FTD symptoms, even within the same family (Kaivorinne et al., 2013, Hsiung et al., 2012, Rohrer et al., 2015a). Cross-sectional studies from ALS specialty clinics found it accounted for 6–8% of non-familial ALS cases, possibly because the gene is not fully penetrant before the ninth decade (Majounie et al., 2012), or because a family history of dementia was not appreciated. Because ALS and FTD patients are typically seen in different specialty clinics, studies of brain imaging have compared C9orf72 ALS and FTD patients to sporadic patients with the same phenotypes. *C9orf72*-specific imaging features of patients with ALS and FTD have been described in comparison to sporadic patients with the same clinical phenotype (Bede et al., 2013a, Byrne et al., 2012, Rohrer et al., 2015b, Whitwell et al., 2015, Bocchetta et al., 2016). Among behavioral variant FTD patients (bvFTD), patients with *C9orf72* mutations had a more diffuse pattern of atrophy than patients with progranulin or *MAPT* mutations (Whitwell et al., 2015, Rohrer and Warren, 2011). Within ALS cohorts, *C9orf72* ALS patients had greater subcortical atrophy compared to sporadic ALS patients (Bede et al., 2013a, Byrne et al., 2012).

It is not clear whether a common imaging signature exists across all *C9orf72* gene carriers. Understanding how the imaging findings differ between clinical phenotypes, and the relationship between disease progression and imaging findings requires a comparison between *C9orf72* gene carriers with different phenotypes. Moreover, reports of imaging differences between asymptomatic *C9orf72* gene carriers and non-carriers (Rohrer et al., 2015b, Walhout et al., 2015) raise the question whether symptoms are preceded by subclinical degeneration or whether differences reflect a developmental feature. If imaging changes in asymptomatic carriers result from ongoing subclinical degeneration, the changes would be expected to progress over time. In symptomatic carriers, the relationship between imaging findings and disease progression is relevant to the question of whether imaging changes can be used as a biomarker to follow disease progression in clinical trials (Cardenas-Blanco et al., 2016, Filippi et al., 2015). The time frame of clinical trials in ALS tends to be short, typically 6 months to one year, because of the short survival after a diagnosis of ALS is made (Miller et al., 1999). For an imaging measure to be useful as a biomarker, it should have measurable changes over 6 months, have good reliability and effect size, and be correlated to a clinical measure of disease activity. To better understand the relationship between the C9orf72 genotype and phenotypes, symptomatic and asymptomatic *C9orf72* gene carriers were recruited for a prospective longitudinal imaging study with clinical evaluations. These participants were not selected for particular clinical presentations in order to permit the spectrum of *C9orf72* phenotypes to be explored. Imaging of *C9orf72* gene carriers was compared to sporadic ALS patients and healthy controls. The relationship between imaging changes and disease activity was evaluated longitudinally, as well as in cross-sectional comparisons to sporadic ALS patients and healthy controls.

## 2 Methods

### 2.1 Subjects

#### 2.1.1 *C9orf72* participants and controls

Twenty-seven unrelated participants with expansion mutations in the *C9orf72* gene (15 males, 12 females; hereafter referred to as C9 + subjects) and 28 healthy controls (18 males, 10 females) gave written, informed consent for participation in protocols that were approved by the NIH Combined Neuroscience Institutional Review Board (NCT01925196; NCT01517087).

#### 2.1.2 Sporadic ALS cohort

Twenty-two patients with no family history of ALS or FTD (11 males, 11 females; hereafter referred to sALS) gave written, informed consent for an imaging and cognitive study approved by the NINDS Institutional Review Board (NCT00334516). This cohort was studied prospectively between 2006 and 2010, prior to the discovery of *C9orf72,* and the approved protocol did not include stored DNA. Clinical characteristics and selected imaging findings from this cohort have been previously described (Kwan et al., 2012).

### 2.2 Diagnostic testing

#### 2.2.1 C9 + subjects

A hexanucleotide repeat expansion of *C9orf72* was confirmed in all C9 + subjects by CLIA-certified clinical testing laboratories. All C9 + subjects were examined by an experienced neurologist (MKF or BT), and underwent electromyography and cognitive testing to determine a clinical diagnosis. C9 + subjects meeting the El Escorial criteria-revised for possible, probable, or definite ALS (Brooks et al., 2000) were classified as having a clinical diagnosis of ALS. Cognitive testing for determining the clinical diagnosis included the Mattis Dementia Rating Scale (DRS-2) (Jurica et al., 2001), which tests attention, initiation-perseveration, conceptualization, construction and memory, and provides age- and education- scaled scores, and the Frontobehavioral Inventory, an examiner-delivered 36-item caregiver questionnaire. (Milan et al., 2008) C9 + subjects meeting the Rascovsky criteria (Rascovsky et al., 2008) for possible, probable or definite bvFTD were classified as having a clinical diagnosis of bvFTD. Some C9 + subjects met criteria for both ALS and bvFTD. As a result, C9 + subjects were classified into four phenotype groups: 1) C9 + asymptomatic, 2) C9 + ALS, 3) C9 + bvFTD and 4) C9 + ALS-FTD.

#### 2.2.2 Healthy controls

All healthy control subjects had a normal neurological examination and normal scores on the Mini-mental state exam. (Folstein et al., 1975) None had a family history of ALS or FTD.

#### 2.2.3 Sporadic ALS

A history and neurological exam was carried out by an experienced neurologist (MKF or JK). All ALS patients underwent diagnostic testing to confirm that they fulfilled the El Escorial criteria for possible, probable, or definite ALS (Brooks et al., 2000). Cognitive testing for determining the clinical diagnosis included the Mattis Dementia Rating Scale (Jurica et al., 2001) and the Frontal Systems Behavioral scale Frontal Systems Behavioral Scale (Stout et al., 2003), which consists of patient and caregiver interview on current and premorbid symptoms of apathy, disinhibition, and executive dysfunction. None of the sALS patients had dementia.

### 2.3 Clinical measures

The severity of motor and executive dysfunction of C9 + subjects and sALS patients was measured at the baseline visit, and at follow-up visits of the C9 + subjects, for comparison with imaging findings. Motor function was measured using the ALS Functional Rating Scale-Revised (ALSFRS-R), a 48-point scale assessing bulbar, fine motor, gross motor, and respiratory function (Cedarbaum et al., 1999). Letter fluency, a measure of executive function, was measured using the fluency subtests of the Delis-Kaplan Executive Function System (Delis et al., 2004). Letter fluency results are reported here as the total correct number of words generated in 1 min for 3 letters, either written or orally. The alternate version of the D-KEFS fluency subtest was used at the 6-month follow-up of C9 + subjects.

### 2.4 Imaging methods

#### 2.4.1 Magnetic resonance imaging (MRI) acquisition

##### 2.4.1.1 C9 + subjects and healthy controls

A 3T MRI scanner with a receive-only, eight-channel head coil (GE HDX, GE Medical Systems, Milwaukee, WI) was used for imaging the C9 + subjects and healthy controls. Two high-resolution T1-weighted sequences (3D fast spoiled gradient echo sequence, TR + TE vary, TI = 450 ms, FOV 240 × 240, Resolution 256 × 256, 140 slices, 1 mm thickness, voxel = 0.9375 mm × 0.9375 mm × 1 mm) were acquired and averaged at each time point for FreeSurfer evaluation of volumes and cortical thickness. All patients undergoing scanning had a vital capacity of at least 60% predicted volume; a pulse oximeter was used to monitor patients with vital capacity below 70%, and all had > 90% oxygen saturation throughout the scanning session.

##### 2.4.1.2 Sporadic ALS cohort

MRI studies of the sALS patients were performed on a 3T scanner (Philips Achieva, Best, the Netherlands) using a receive-only, eight-channel head coil. For volumetric and thickness measurements, a high resolution T1 weighted image (3D turbo field echo sequence, TR = 8.6 ms, TE = 3.9 ms, TI = 700 ms, FOV 240 × 240, Resolution 256 × 256, 140 slices, 1 mm thickness, voxel = 0.9375 mm × 0.9375 mm × 1 mm) was obtained. All patients had vital capacity of 70% or greater.

#### 2.4.2 Image processing: Volumetric and cortical thickness measures

Cortical reconstruction and volumetric segmentation was performed with the FreeSurfer image analysis suite (http://surfer.nmr.mgh.harvard.edu/). The technical details of these procedures have been described and validated in prior publications (Fischl et al., 1999, Fischl et al., 2002, Fischl et al., 2004). Briefly, the processing included skull stripping, Talairach transformation, optimization of the grey matter-white matter and grey matter-CSF boundaries, segmentation, and tessellation (Dale et al., 1999). Images were visually inspected and scans with errors in segmentation of cortical or CSF surfaces and grey-white junctions were manually corrected. Atrophied brains were carefully inspected for errors. The tessellated surfaces were then inflated and registered to the Desikan-Killiany atlas which parcellates the cerebral cortex into 34 regions in each hemisphere based on gyral and sulcal structures (Desikan et al., 2006). The FreeSurfer longitudinal stream, which creates an unbiased within-subject template for measuring changes in cortical thickness over time (http://surfer.nmr.mgh.harvard.edu/fswiki/LongitudinalProcessing) (Reuter et al., 2012) was used. Measures of regional cortical thickness were compared between subgroups and longitudinally. The FreeSurfer QDEC tool was used to carry out whole-brain vertex-wise comparisons between the baseline scans of symptomatic C9 + subjects and healthy controls, and between the C9 + ALS/ALS-FTD patients and sALS patients, including gender as a covariate. Data were smoothed at 10 mm, and p < 0.05 with FDR correction was used as criteria for significance.

The volume of ventricles and subcortical structures were also obtained from FreeSurfer. The ventricular volume was obtained by summing the number of voxels within all ventricles. Volumes were expressed as a percentage of total intracranial volume (%TIV) to account for differences in head size for group comparisons. Because FreeSurfer is not optimized to produce an accurate measure of TIV, this value was calculated in Statistical Parametric Mapping 12 (SPM12, method of Malone and colleagues (Malone et al., 2015).

#### 2.4.3 Statistics

Demographic and clinical results are reported as means ± standard deviations (SD) in tables and text. The Shapiro-Wilk test was used to test normality of clinical variables. Age, disease duration, and letter fluency scores were normally distributed, and comparisons between groups were carried out by ANOVA or *t*-tests. The ALSFRS-R was not normally distributed. Mann-Whitney or Kruskal Wallis tests were used for variables that were not normally distributed. ANCOVA was used to compare volumetric and regional thickness measures, with age and gender as covariates. Post-hoc testing between C9 + subgroups and controls was carried out using Dunnett's test for multiple comparisons or Dunn's correction for nonparametric testing. Pearson's r was used to assess correlations. A repeated measures ANOVA was used to assess longitudinal changes, adjusting for age and gender as covariates. Statistics were calculated with SPSS v.23 (IBM SPSS Statistics). Significance was defined as p < 0.05, corrected for multiple comparisons. G*Power v 3.1 (http://www.gpower.hhu.de/en.html, accessed 8/25/2016) was used for power and sample size calculations.

## 3 Results

### 3.1 Clinical demographics

#### 3.1.1 Demographics of groups

There was no difference in age between the C9 +, sALS, and healthy control groups. The ratio of men to women was similar in the groups, with slightly more men than women in the C9 + and control groups (Table 1). Symptom duration and ALSFRS-R did not differ between sALS and symptomatic C9 + patients.

#### 3.1.2 C9 + subgroup demographics

At the baseline visit, seven C9 + subjects were asymptomatic, eleven had C9 + ALS, three had C9 + bvFTD, and six had C9 + ALS-FTD (Table 1). In the C9 + subgroups, asymptomatic subjects were younger than C9 + subjects with ALS-FTD (p = 0.013). In symptomatic C9 + subjects, cognitive-behavioral changes were the first symptom in six patients and a motor deficit was the first symptom in the other 14, including five patients with bulbar-onset ALS. Age of symptom onset and disease duration did not differ among subgroups of symptomatic C9 + patients. Cognitive testing showed that letter fluency and the DRS-2 age- and education-scaled score were not significantly different between the sALS and the C9 + groups as a whole (*t*-test, p = 0.1273, p = 0.6380). Asymptomatic C9 + subjects had significantly better scores than C9 + ALS-FTD and bvFTD patients, but were not different from C9 + ALS subjects.

Nineteen C9 + subjects returned for a 6-month follow-up (mean = 5.7 ± 0.7 months), and seven returned for an 18-month follow-up. The 6-month follow-up cohort consisted of five C9 + asymptomatic, six C9 + ALS, five C9 + ALS-FTD, and three C9 + bvFTD subjects. Twenty-three of the 28 healthy controls had two imaging sessions and seventeen had three imaging sessions at various intervals. Control scans were selected with a mean interval of 5.6 ± 4.3 months to compare with the 6-month follow-up scans of C9 + subjects.

### 3.2 Volumetric measures

#### 3.2.1 Baseline differences in ventricular volume

**Fig. 1:**
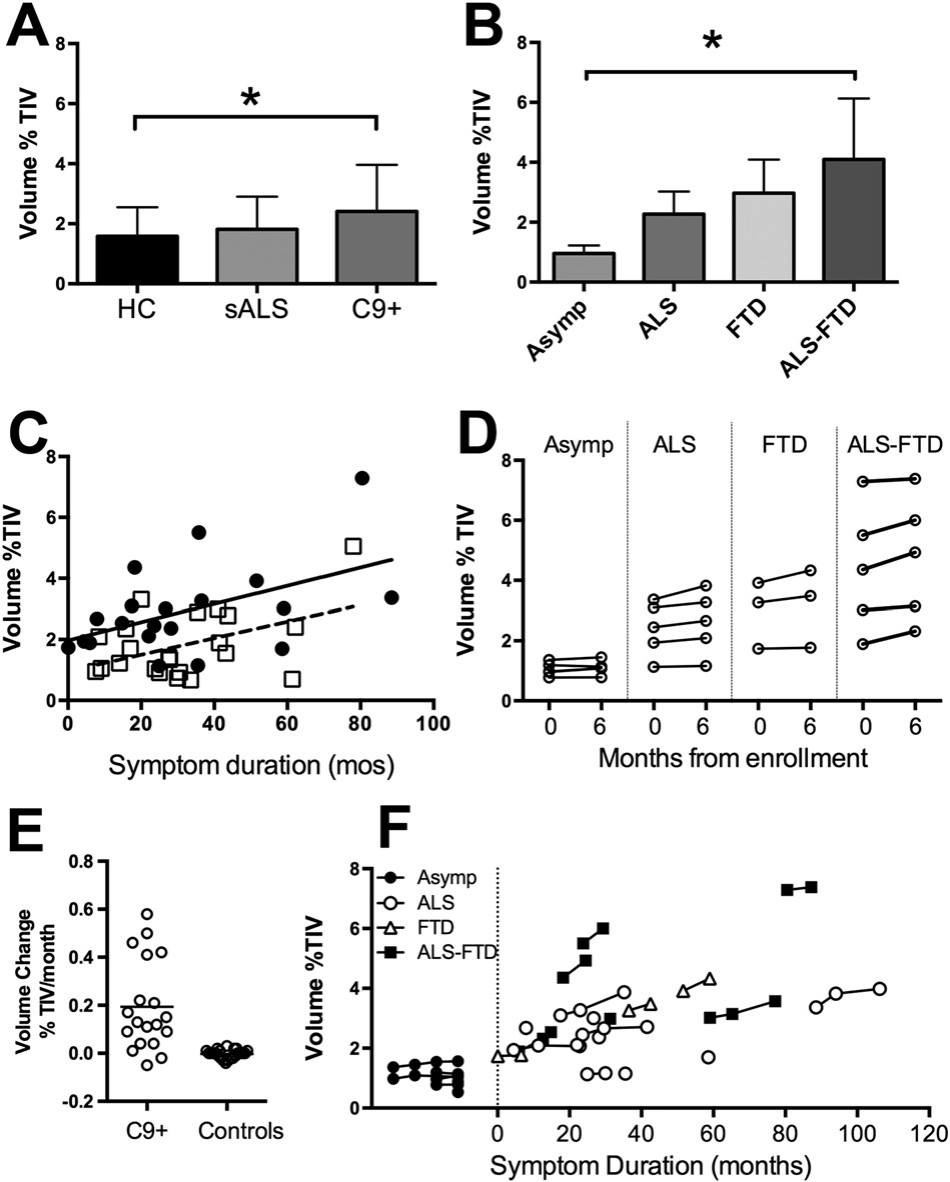
Ventricular volumes expressed as the percent of Total Intracranial Volume (TIV) at baseline (A–C) and longitudinally (D–F). A) At baseline, C9 + subject group had larger ventricles than healthy controls (HC; *p < 0.05). Sporadic ALS (sALS) did not differ from controls. B) Within the C9 + subgroups, C9 + ALS-FTD patients had larger ventricles than asymptomatic C9 + carriers (Asymp) and HC (*p < 0.05). C) Ventricular volume increased with disease duration in C9 + subjects (filled circles, solid lines) and sALS (open squares, dashed line) with a similar slope, but C9 + had larger ventricles, adjusted for age, compared to sALS and HC. D) Change in ventricular volume over 6 months in C9 + subjects. E) Rate of ventricular enlargement per month, calculated from baseline to 6 month-follow-up of 19 C9 + subjects and 23 HC (individual values, line indicates group mean). F) Ventricular volumes all scans C9 + subjects, including longitudinal scans for asymptomatic C9 + (filled circles), C9 + ALS (open circles), C9 + bvFTD (open triangles) and C9 + ALS-FTD (filled squares) plotted by symptom duration.

At the baseline visit, the C9 + subject group as a whole had evidence of brain atrophy, with increased ventricular volume compared to controls (Fig. 1A). The ventricular volume of the sALS group did not differ from controls. Among the C9 + subjects, the C9 + subgroup with ALS-FTD had larger ventricles than C9 + asymptomatic subjects and healthy controls (Fig. 1B). In all groups, age was correlated with ventricular volume, with no difference in slope between groups, although C9 + subjects had a significant intercept shift relative to controls and sALS, indicating greater atrophy for age. Ventricular volume was also correlated with symptom duration, with a significant shift in the intercept in the C9 + group (Fig. 1C). There was no significant effect of gender on ventricular volume, which was expressed as percentage of total intracranial volume.

#### 3.2.2 Longitudinal changes in ventricular volume

The ventricular volume increased on average by 3.5 ml in the symptomatic C9 + subjects in the 6-month interval between scans (Fig. 1D). The age-adjusted rate of ventricular enlargement was greater in C9 + subjects as a whole than controls, with a relatively broad range (Fig. 1E). Post-hoc statistics identified only the C9 + bvFTD and C9 + ALS-FTD subgroups as having greater ventricular enlargement than healthy controls over 6 months. In asymptomatic C9 + subjects, ventricular volume was relatively stable, including over longer longitudinal intervals, whereas symptomatic C9 + patients had increasing ventricular volumes, even among the symptomatic patients with long symptom duration (Fig. 1F).

#### 3.2.3 Volumes of subcortical structures

In scans obtained at the baseline visit, the volume of the thalamus was smaller in C9 + subjects compared to controls and sALS patients. Both right and left sides of the thalamus had smaller volumes. Post-hoc testing showed that the thalamic volumes of C9 + bvFTD and C9 + ALS-FTD subgroups were less than controls (Table 2). There was no significant difference in the volume of the cerebellum, caudate, putamen, or pallidum among the C9 +, sALS, and control groups at baseline. Over the 6-month follow-up interval, C9 + subjects had significant volume loss in the thalamus but not in the other subcortical regions compared to controls.

#### 3.2.4 Volume of cortical white matter

The volume of cortical white matter of the right and left hemispheres was less for C9 + subjects than sALS patients and controls (left cortex p = 0.014; right cortex p = 0.020), with age included as a covariate. Gender and disease duration were not significant covariates. Dunnett's test showed that among the C9 + subgroups, the C9 + ALS-FTD and FTD white matter volumes differed from that of sALS patients (p < 0.05). The white matter volume of sALS patients did not differ from controls (left, p = 0.843; right, p = 0.891).

### 3.3 Cortical thickness

#### 3.3.1 Vertex-wise comparison between groups

**Fig. 2:**
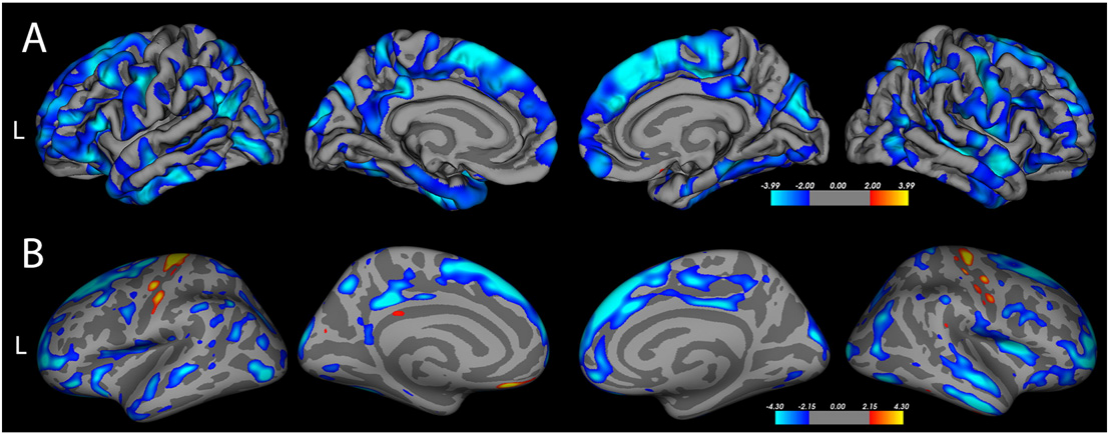
Regions of cortical thinning in whole-brain analyses of C9 + subjects compared to other groups are indicated in blue. A) Symptomatic C9 + subjects (n = 20) have diffuse patchy thinning, with broad swaths of thinning in frontal and temporal regions compared to healthy controls (n = 28). B) C9 + patients with ALS or ALS-FTD (n = 17) have patchy regions of thinning with a frontal predominance compared to sporadic ALS patients (n = 22). In contrast, sALS patients have thinner motor cortex than C9 + patients, best seen in this inflated view (yellow-orange areas). Vertex-wise comparison using FreeSurfer. (Fischl and Dale, 2000) The color scale indicates the negative log of the p values (threshold p = 0.05, FDR corrected).

A whole-brain comparison of cortical thickness between the 20 symptomatic C9 + subjects versus the 28 healthy controls showed a widespread, patchy distribution of cortical thinning (Fig. 2 A). Clusters of thinning were particularly prominent in the superior and medial frontal cortex and temporal poles bilaterally. Thinning also occurred in the cuneus, posterior cingulate and small scattered cortical regions. Areas of sparing included the occipital pole and the anterior and middle portions of the cingulate gyrus. There was no difference in cortical thickness between C9 + asymptomatic subjects and controls. When the group of 17 C9 + ALS and ALS-FTD patients were compared to the group of 22 sALS patients, C9 + ALS patients had thinner cortex in many areas, particularly in frontal and temporal regions (Fig. 2 B). Interestingly, the motor cortex was less thin (red, yellow) in C9 + ALS and ALS-FTD patients than in sALS patients.

#### 3.3.2 Regional differences in cortical thickness

The mean cortical thickness of both left and right hemispheres was less in the C9 + subject group as a whole compared to healthy controls and to sALS patients in the baseline scans (Table 3). Among the C9 + subgroups, the mean cortical thickness was less in the C9 + ALS-FTD and C9 + ALS patients compared to controls. To determine which cortical areas most accounted for differences in the mean hemispheric thickness, a multivariate analysis was carried out on the 34 gyral regions of each hemisphere (Desikan et al., 2006) including age and gender as covariates. As predicted from the whole-brain vertex-wise analysis, the C9 + group had thinning in several non-motor regions compared to sALS patients and controls, particularly frontal regions. Regions with differences between groups are indicated by letters in Table 3. Both sALS and C9 + groups had thinning of the right precentral gyrus. The thickness of the left precentral and paracentral gyri, the insula, and the cingulate cortex was not significantly different between C9 +, sALS, and control groups.

Cortical thickness of the regions that differed significantly from controls were compared between the subgroups of C9 + subjects and the sALS patients to look for differences associated with genotype. C9 + ALS patients had significant thinning of the superior frontal and orbitofrontal cortex bilaterally compared to sALS patients (Dunnett's test; p < 0.05). These regions were also thinner in the C9 + ALS-FTD and C9 + FTD subgroups than in sALS patients. The C9 + ALS-FTD and C9 + FTD subgroups also had thinning of several frontal and temporal regions, of which several were adjacent, compared to sALS patients (Supplemental Table 1). Asymptomatic C9 + subjects did not have any regions of thinning compared to sALS patients.

#### 3.3.3 Longitudinal changes in thickness

Changes in regional cortical thickness between the baseline and 6-month follow-up scan were compared for the 19 C9 + subjects and the group of 23 healthy controls. The C9 + subject group included 5 asymptomatic subjects, 6 C9 + ALS, 5 C9 + ALS-FTD, and 3 C9 + bvFTD patients. Although the inter-scan intervals of C9 + group and control group were matched between groups, rates of thinning were analyzed to adjust for variation in inter-scan intervals. The rate of cortical thinning did not differ between healthy controls and the C9 + group as a whole or the C9 + subgroups.

### 3.4 Clinical correlations

#### 3.4.1 Cortical thickness

Cortical thickness and letter fluency correlated with age, although the ALSFRS-R score did not. Therefore, age was included as a covariate, and partial correlations were calculated. There was a weak but significant correlation between the ALSFRS-R of the C9 + and sALS groups with the thickness of the precentral gyri bilaterally (right side r = 0.389, p = 0.007; left side r = 0.394, p = 0.006). The ALSFRS-R was not significantly correlated with thickness of other cortical regions. In contrast, the letter fluency score was correlated with the age-adjusted thickness of many cortical areas, with moderately strong correlations with the caudal middle frontal gyrus and pars opercularis bilaterally, the right pars orbitalis, and left entorhinal cortex (r = 0.5–0.7). Several other frontal, temporal, and parietal regions exhibited weaker correlations (r = 0.3–0.5) as did the precentral and paracentral gyri (Supplemental Table 2).

#### 3.4.2 Volumes

The ALSFRS-R scores were not correlated with the baseline ventricular volume or volumes of subcortical structures of sALS and C9 + subjects. There was a weak correlation between the longitudinal change in ALSFRS-R and the change in ventricular volume (r = 0.489, p = 0.034) but significance did not survive correction for age. At baseline, letter fluency scores had a weak negative correlation with ventricular volume (r = − 0.352, p = 0.028) and a weak positive correlation with the volume of the thalamus (r = 0.446, p = 0.004) adjusted for age and gender.

### 3.5 Reproducibility of imaging measures with repeat scanning

In healthy controls, imaging measures of ventricular and all subcortical volumes were strongly correlated between scans at the 2 time points (Pearson's correlation coefficients: ventricular volume, r = 0.997; thalamus, r = 0.994; cerebellum, r = 0.929; caudate r = 0.986; putamen r = 0.971, pallidum 0.932. All p values < 0.0001). The measure of ventricular volume, expressed as %TIV, was highly reproducible in the two scans (Lin's concordance correlation coefficient (ρ_c_) = 0.9967). These data show that ventricular volume can be reliably measured in repeat scans from healthy controls who do not have ongoing degeneration. The healthy control group had minimal change in ventricular volume over 6 months (mean difference of − 0.22 ml/6-month), an order of magnitude less than symptomatic C9 + subjects. Although this finding may seem encouraging that the measurement of ventricular volume is sufficiently reliable to follow over time as a biomarker of progression, a power calculation based on data from the 14 symptomatic subjects with longitudinal imaging shows that relatively large sample sizes would be needed to detect the effects of an intervention between treated and untreated groups of C9 + symptomatic subjects. Using the measures of ventricular volume change in symptomatic C9 + subjects in this study, Table 4 shows the sample and effect sizes calculated for hypothetical interventions that reduce ventricular volume loss by 20–50% compared to no treatment. Detecting even a medium effect (0.5 < d > 0.3), would require sample sizes of 50 or more patients per group. This reflects, in part, the large variability between C9 + subjects.

Regional measures of cortical thickness were also reproducible between the 2 scans, although correlations were weaker than for volume measures. For the 34 cortical regions of each hemisphere, thickness of each region at the two time points was strongly correlated and significant (r > 0.7, p < 0.0001), except for two regions, the right precuneus and right medial orbitofrontal cortex. For the other areas, correlation coefficients ranged from 0.748 to 0.966. Eighteen regions of the left hemisphere and 14 regions of the right hemisphere had very strong correlations (r > 0.9) between the two time points, including regions that would be of particular interest, such the precentral gyrus and several frontal and temporal regions (Supplemental Table 3).

## 4 Discussion

Persons with an expansion mutation of the *C9orf72* gene had diffuse brain atrophy compared to healthy controls and sporadic ALS patients. Evidence of cortical and subcortical atrophy was seen in symptomatic *C9orf72* carriers, regardless of whether their clinical phenotype was ALS, FTD, or ALS-FTD. Among the C9 + phenotypes, atrophy was most severe in C9 + patients with clinical features of bvFTD. However, even C9 + ALS patients without dementia had regions of thinning compared to sporadic ALS patients. Atrophy was the common underlying feature of disease associated with *C9orf72* mutations. Because we did not detect significant atrophy in the asymptomatic C9 + subjects in this study, it is less likely to reflect a developmentally acquired condition. Over follow-up intervals up to 18 months, ventricular volumes remained stable in the clinically-stable asymptomatic C9 + subjects, whereas progressive ventricular enlargement was seen in symptomatic C9 + subjects. For this reason, we suggest that atrophy is directly associated with active disease, and that longitudinal imaging may reveal progression of disease.

In sporadic bvFTD and sporadic ALS, pathological studies have led to the hypothesis that degeneration spreads from one region to another in a disease-specific pattern: progressing from rostral to caudal from the frontal cortex in bvFTD (Brettschneider et al., 2014, Kim et al., 2012) and spreading centrifugally from the motor cortex in sporadic ALS (Braak et al., 2013). Cross-sectional imaging studies of patients with sporadic ALS (Agosta et al., 2012, Roccatagliata et al., 2009, Verstraete et al., 2012, Kassubek et al., 2014) and sporadic FTD (Mahoney et al., 2012, Rohrer and Rosen, 2013, Huey et al., 2009), comparing patients at different stages of disease, have largely been consistent with these pathological hypotheses. A longitudinal study of sporadic ALS patients was also consistent, finding local grey matter loss in motor cortex earlier than in regions receiving motor cortical inputs, such as the frontal cortex and basal ganglia (Menke et al., 2014). Disease-specific patterns were not evident in the C9 + patients in this study. The C9 + clinical phenotypes were not differentiated by localized thinning or atrophy, but had thinning across many scattered cortical regions. In most patients, right and left sides were strongly correlated, without lateralized atrophy, as occurs in FTD patients with mutations in the *GRN* gene (Rohrer et al., 2015a). These findings suggesting a more diffuse spread of degeneration in *C9orf72* related diseases.

The findings of atrophy in symptomatic patients agree with earlier studies that compared groups of patients with sporadic ALS or FTD to *C9orf72* patients with the same phenotype (Bede et al., 2013a, Byrne et al., 2012, Rohrer et al., 2015b, Whitwell et al., 2015). As in previous studies, cognitive impairment was associated with subcortical atrophy, notably thalamic atrophy, in *C9orf72* ALS patients (Bede et al., 2013a, Bede et al., 2013b). The C9 + subjects with ALS-FTD and bvFTD in this study had smaller thalamic volumes, consistent with reports from other FTD patient populations (Rohrer et al., 2015a), as well as patchy cortical thinning. Letter fluency, the measure of executive function used in this study, has been shown to activate the left temporal lobe and several frontal regions in task-based fMRI (Cook et al., 2014, Abrahams et al., 2004). However, we found that letter fluency scores were correlated with cortical thinning in many regions, including the motor cortex. This may reflect the ease of disrupting cognitive networks for searching and retrieving words by letter. This study and others (Machts et al., 2015) highlight the importance of characterizing both motor and cognitive-behavioral function in patients with *C9orf72* mutations. In this study, in which all C9 + subjects underwent the same testing for motor and cognitive function, a third of the C9 + subjects in this study met clinical criteria for both ALS and bvFTD. It is unknown whether the proportion of phenotypes in this sample is representative of their proportions in the population of *C9orf72* mutation carriers. Population-based studies fully characterizing motor and cognitive symptoms would be needed to determine the relative proportions of each phenotype among the population of C9orf72 carriers. In one large US ALS clinic, 61 of 781 ALS patients carried the C9orf72 mutation (7.8%), and of these nine (14.8%) had comorbid FTD (Umoh et al., 2016). We had a higher proportion of co-morbid C9 + ALS and FTD in our study. This may reflect that patients whose symptoms begin with cognitive-behavioral symptoms, and later develop motor symptoms, are not represented in an ALS clinic population. Recruitment for this study reached out to memory clinics as well as ALS specialty clinics for referral of patients with *C9orf72* mutations. We recognize as well that the patients in this study represent a selected group, willing to travel to participate in research.

The longitudinal symptomatic C9 + patients showed considerable variability in clinical progression, with symptom durations ranging from 1 month to 88 months at the time of enrollment. A relatively indolent course prior to enrollment was present in one C9 + ALS patient as well as in several C9 + ALS-FTD patients whose first symptom was cognitive impairment. One limitation in evaluating the relationship between imaging findings and symptom duration was difficulty in pinpointing the onset of cognitive-behavioral changes. Additionally, some C9 + ALS patients who did not meet criteria for bvFTD may have had milder degrees of cognitive and behavioral impairment. The small sample size did not allow further gradations of cognitive impairment. Drop-out of rapidly progressing patients from 6-month follow-up scans is another potential source of bias. A 6-month follow-up interval was selected because it is a time span over which ALS patients deteriorate, and is also a typical time span for interventional clinical trials. However, because some rapidly progressing ALS patients were unable to return for scanning, the longitudinal cohort was enriched for asymptomatic C9 + and less impaired patients. More severely affected patients may have greater imaging changes, and thus the changes reported here may be an underestimate.

Understanding whether imaging provides genotype- or phenotype-specific signatures, and the temporal relationship between imaging findings and clinical symptoms, is important to understand in a familial disease. Familial motor neuron disorders present an opportunity to identify imaging changes associated with early disease activity in presymptomatic gene carriers (Benatar and Wuu, 2012, Turner and Benatar, 2015). Studies in familial FTD patients identified changes in asymptomatic carriers who were 5–10 years younger than the typical age of disease onset in their family members (Rohrer et al., 2015b, Dopper et al., 2014). Similarly, metabolic changes have been identified in the spinal cord of asymptomatic carriers of the SOD1A mutation (Carew et al., 2011a). A study of asymptomatic *C9orf72* gene carriers and non-carriers within a single large family found grey matter thinning in several cortical regions (Walhout et al., 2015). It is unclear whether genotype-specific imaging findings herald the onset of clinical disease. Although our study was unable to identify structural changes in asymptomatic carriers compared to healthy controls, longitudinal follow-up is needed to understand whether slowly developing changes occur, and whether imaging differences predict the development of clinical symptoms.

In this study, we analyzed ventricular volume as a candidate biomarker for a number of reasons: enlarged ventricles are a visible abnormality seen in clinical scans, the scan-rescan reproducibility of ventricular volume with our methods was excellent for healthy controls, and ventricular size can be measured using a variety of different software programs. Moreover, ventricular enlargement may pick up atrophy from patchy cortical thinning, white matter atrophy, or mild subcortical atrophy when affected regions vary between individuals. Our finding of ventricular enlargement in the symptomatic C9 + group over 6 months is consistent with previous longitudinal studies in sporadic ALS that detected grey matter loss over a 6-month (Menke et al., 2014) or 1-year (Kwan et al., 2012) interval as well as a study that found annualized rates of atrophy in the range of 2–3% in bvFTD groups (Whitwell et al., 2015). While detecting changes in C9 + symptomatic patients over 6 months offers encouragement that imaging may be useful to follow disease progression in individuals within the time window of a typical therapeutic trial, the variability among individuals would require large sample sizes if ventricular enlargement were to be used as a sole biomarker. The variability might be improved with a better understanding of factors contributing to individual differences in disease onset and progression. Regardless, the change in ventricular volume may be useful to create a composite marker in combination with other measures. These could include other imaging modalities, such as diffusion imaging (Cardenas-Blanco et al., 2016, Muller et al., 2016) or spectroscopy (Carew et al., 2011b, Stagg et al., 2013), or biofluid markers such as the *C9orf72* repeat-associated dipeptide (Su et al., 2014).

Why *C9orf72* mutations cause different phenotypes, even within the same family (Byrne et al., 2012) is not clear. Different phenotypes could result from distinct disease initiation sites that spread through synaptic connections, or from patient-specific vulnerabilities of different neurons to the molecular pathways disturbed by the *C9orf72* mutation (Donnelly et al., 2013, Ling et al., 2013). Alternatively, *C9orf72* mutations could be causing degeneration randomly in individual neurons throughout the brain, such that clinical symptoms become evident when brain networks fail (Schmidt et al., 2016, Schmidt et al., 2014, Lee et al., 2014). Whether the phenotypic variability of subjects with the *C9orf72* mutation can be linked to selective vulnerability or patterns of spread through axonal networks, and how these might interact with genetic (van Blitterswijk et al., 2014) and epigenetic (McMillan et al., 2015) modifiers of clinical phenotypes of *C9orf72*, will need to be assessed in C9 + subjects scanned before and soon after onset of clinical symptoms. Studies of a larger numbers of *C9orf72* carriers imaged before and after the onset of symptoms are needed to test whether disease spreads through networks or occurs randomly across the brain.

## 5 Conclusion

Symptomatic carriers of the *C9orf72* expansion mutation exhibit more brain atrophy than healthy controls of a similar age. Atrophy occurs regardless of whether carriers present with an ALS or bvFTD clinical phenotype, although atrophy is greater in patients with cognitive-behavioral impairment. Ventricular enlargement and thalamic atrophy can be detected in groups of symptomatic patients over a 6-month interval.

The following are the supplementary data related to this article.

Supplemental Table 1 Thickness of cortical regions at baseline scans. Supplemental Table 1

Supplemental Table 2 Age-adjusted correlations of ALSFRS-R and Letter Fluency scores with regional cortical thickness. Supplemental Table 2

Supplemental Table 3 Cortical regions with highly reproducible scan-rescan thickness measures (r > 0.90) in healthy controls. Supplemental Table 3

## Figures and Tables

**Table 1:** Demographics of subject groups, baseline evaluation.

	Controls	sALS patients	*C9orf72* subjects (all)	C9 + diagnosis subgroups			
				Asymptomatic	ALS	ALS-FTD	bvFTD
Number	28	22	27	7	11	6	3
Age (years)	52.8 ± 9.1	55.9 ± 11.1	52.2 ± 11.7	41.2 ± 11^a^	52.3 ± 8.8	60.8 ± 10.2	60.6 ± 5.1
Male:Female	18:10	11:11	15:12	1:6	5:6	6:0	3:0
Symptom Duration* (months)	–	27.1 ± 17.4	33.7 ± 24.0	–	30.7 ± 23.9	33.7 ± 29.3	29.4 ± 26.5
ALSFRS-R	48	40.0 ± 6.9	41.6 ± 7.6	48	36.2 ± 8.4^b^	41.8 ± 5.26^b^	46.3 ± 2.9
MMSE	29.0 ± 1.2						
DRS-2 age/educ Scaled score	–	8.3 ± 5.1	7.6 ± 4.3	10.7 ± 2.9^a^	8.6 ± 3.6	3.8 ± 3.8	2.3 ± 2.3
Letter fluency (total for 3 letters)	–	35.4 ± 11.8	28.6 ± 14.4	42.3 ± 12.4^a^	30.0 ± 11.2	13.8 ± 3.0	16.0 ± 4.4

*Excluding asymptomatic carriers; MMSE mini-mental state exam; DRS-2 — Mattis Dementia Rating Scale, scaled for age and education, normal mean = 10; within C9 + subgroups.

a Significantly different from C9 + ALS-FTD and bvFTD subgroups.

b Significantly different from C9 + asymptomatic subjects.

**Table 2:** Volume of subcortical structures.

	Healthy controls	sALS	C9 + (all)	C9 + subtypes			
				Asymp.	ALS	bvFTD	ALS-FTD
Cerebellum	8.65 ± 0.77	8.28 ± 0.91	8.46 ± 0.91	9.22 ± 0.74	8.64 ± 0.72	7.94 ± 0.36	7.53 ± 0.65
Thalamus	0.98 ± 0.11	0.91 ± 0.10	0.88 ± 0.12^a^	1.01 ± 0.08	0.90 ± 0.07	0.76 ± 0.09^a^	0.77 ± 0.09^a^
Caudate	0.50 ± 0.06	0.51 ± 0.06	0.47 ± 0.08	0.49 ± 0.07	0.52 ± 0.07	0.38 ± 0.07	0.41 ± 0.05
Putamen	0.72 ± 0.10	0.70 ± 0.11	0.67 ± 0.15	0.78 ± 0.09	0.72 ± 0.11	0.51 ± 0.12	0.53 ± 0.10
Pallidum	0.20 ± 0.03	0.21 ± 0.03	0.19 ± 0.04	0.21 ± 0.03	0.21 ± 0.03	0.14 ± 0.02	0.15 ± 0.03

Volumes are expressed as % of total intracranial volumes. sALS = sporadic ALS, Asymp = C9 + asymptomatic carriers.

a Less than controls, p < 0.05, Dunnett's test, corrected for multiple comparisons.

**Table 3:** Thickness of selected cortical regions at the baseline scan.

	Left hemisphere				Right hemisphere			
	Healthy controls (n = 28)	C9 + subjects (all) (n = 27)	Sporadic ALS (n = 22)	Sig	Healthy controls (n = 28)	C9 + subjects (all) (n = 27)	Sporadic ALS (n = 22)	Sig
Mean thickness (hemisphere)	2.45 ± 0.09	2.33 ± 0.16	2.44 ± 0.11	A, B	2.44 ± 0.09	2.33 ± 0.17	2.43 ± 0.12	A, B
Precentral	2.56 ± 0.17	2.44 ± 0.21	2.40 ± 0.16		2.55 ± 0.16	2.40 ± 0.23	2.34 ± 0.19	B, C
Paracentral	2.44 ± 0.14	2.32 ± 0.18	2.37 ± 0.23		2.43 ± 0.14	2.28 ± 0.18	2.39 ± 0.20	
Postcentral	2.12 ± 0.12	2.05 ± 0.16	1.99 ± 0.13		2.10 ± 0.14	2.01 ± 0.21	1.97 ± 0.16	
Insula	2.95 ± 0.16	2.88 ± 0.21	2.94 ± 0.16		2.91 ± 0.15	2.83 ± 0.21	2.92 ± 0.18	
Lateral orbitofrontal	2.45 ± 0.12	2.38 ± 0.21	2.60 ± 0.12	A	2.41 ± 0.11	2.34 ± 0.18	2.49 ± 0.16	A, C
Medial orbitofrontal	2.37 ± 0.12	2.35 ± 0.21	2.35 ± 0.19		2.30 ± 0.12	2.26 ± 0.18	2.48 ± 0.17	A
Rostral middle frontal	2.30 ± 0.09	2.18 ± 0.18	2.32 ± 0.10	A, B	2.25 ± 0.09	2.16 ± 0.17	2.32 ± 0.13	A
Superior frontal	2.59 ± 0.09	2.47 ± 0.23	2.70 ± 0.13	A	2.56 ± 0.10	2.42 ± 0.19	2.68 ± 0.15	A, B
Caudal middle frontal	2.47 ± 0.09	2.35 ± 0.19	2.49 ± 0.12	A, B	2.45 ± 0.11	2.38 ± 0.19	2.44 ± 0.15	
Lateral occipital	2.20 ± 0.11	2.07 ± 0.14	2.20 ± 0.15		2.24 ± 0.13	2.14 ± 0.15	2.27 ± 0.14	A
Fusiform	2.74 ± 0.14	2.61 ± 0.21	2.65 ± 0.15		2.73 ± 0.15	2.58 ± 0.21	2.64 ± 0.14	B
Middle temporal	2.84 ± 0.16	2.72 ± 0.22	2.84 ± 0.20		2.80 ± 0.14	2.71 ± 0.20	2.88 ± 0.19	A
Inferior parietal	2.42 ± 0.10	2.30 ± 0.16	2.45 ± 0.16	A, B	2.42 ± 0.13	2.32 ± 0.18	2.43 ± 0.14	

Means ± SD. Sig = significant differences, corrected for age and multiple comparisons p < 0.05.

A: C9 + < sALS, B: C9 + < HC, C: sALS < HC.

**Table 4:** Sample sizes calculated to detect a 6-month difference in ventricular volume between a group of treated and untreated symptomatic C9 + subjects.

	6-month mean ventricular volume change (ml)	Effect size (Cohen's d)	Sample per group power = 0.8	Sample per group power = 0.9
Current data: Symptomatic C9 +	− 3.5			
20% Improvement	− 2.78	0.26	232	310
30% Improvement	− 2.43	0.41	95	126
40% Improvement	− 2.08	0.57	49	65
50% Improvement	− 1.74	0.74	30	39
