# Supplementary material for: Longitudinal imaging in C9orf72 mutation carriers: Relationship to phenotype
Source: Neuroimage Clin. 2016 Oct 22;12:1035–43. doi: 10.1016/j.nicl.2016.10.014 (PMC5153604; doi:10.1016/j.nicl.2016.10.014)
Supplement: Supplemental Table 1 — Thickness of cortical regions at baseline scans. [file mmc1.docx]

Supplemental Table 1. Thickness of cortical regions at baseline

| **Right hemisphere** | | Healthy controls  N=28 | | Sporadic ALS  N=22 | | All C9+  N= 26 | C9+ subgroups | | | | | | | |
| --- | --- | --- | --- | --- | --- | --- | --- | --- | --- | --- | --- | --- | --- | --- |
|  |  |  |  |  |  |  | C9+ Asymptomatic  N=7 | | C9+ ALS  N=11 | | C9+ FTD  N=3 | | C9+ ALS-FTD  N=5 | |
| Caudal anterior cingulate | | 2.55 ± 0.22 | | 2.60 ± 0.29 | | 2.54 ± 0.23 | 2.54± 0.23 | | 2.52 ± 0.27 | | 2.42 ± 0.15 | | 2.66 ± 0.20 | |
| Isthmus cingulate | | 2.45 ± 0.23 | | 2.42 ± 0.20 | | 2.36 ± 0.24 | 2.45 ± 0.23 | | 2.39 ± 0.19 | | 2.34 ± 0.39 | | 2.20 ± 0.25 | |
| Posterior cingulate | | 2.51 ± 0.20 | | 2.52± 0.18 | | 2.40 ± 0.16 | 2.47 ± 0.16 | | 2.41 ± 0.15 | | 2.28 ± 0.20 | | 2.33 ± 0.14 | |
| Rostral anterior cingulate | | 2.78 ± 0.24 | | 2.78 ± 0.26 | | 2.68 ± 0.22 | 2.75 ± 0.26 | | 2.67 ± 0.26 | | 2.65 ± 0.16 | | 2.64 ± 0.05 | |
| Caudal middle frontal | | 2.45 ± 0.11 | | 2.44 ± 0.15 | | 2.38 ± 0.19 | 2.53 ± 0.13 | | 2.42 ± 0.13 | | 2.24 ± 0.16 | | 2.17 ±0.17 | |
| Frontal pole | | 2.62 ± 0.20 | | 2.68 ± 0.32 | | 2.47 ± 0.25 | 2.49 ± 0.33 | | 2.49 ± 0.17 | | 2.47 ± 0.46 | | 2.39 ± 0.16 | |
| **Lateral orbitofrontal** | | **2.41 ± 0.11** | | **2.49 ± 0.16** | | **2.35 ± 0.18^b^** | **2.48 ± 0.17** | | **2.37 ± 0.10** | | **2.22 ± 0.13 ^d^** | | **2.23 ± 0.25 ^d^** | |
| **Medial orbitofrontal** | | **2.30 ± 0.12** | | **2.48 ± 0.17** | | **2.26 ± 0.18^a, b^** | **2.29 ± 0.17** | | **2.30 ± 0.14 ^d^** | | **2.13 ± 0.10 ^d^** | | **2.22 ± 0.31 ^d^** | |
| Pars opercularis | | 2.49 ± 0.13 | | 2.47 ± 0.15 | | 2.39 ± 0.20 | 2.54 ± 0.16 | | 2.40 ± 0.11 | | 2.29 ± 0.20 | | 2.24 ± 0.28 | |
| Pars orbitalis | | 2.60 ± 0.15 | | 2.67 ± 0.19 | | 2.50 ± 0.23 | 2.64 ± 0.12 | | 2.59 ± 0.11 | | 2.38 ± 0.21 | | 2.20 ± 0.29 | |
| Pars triangularis | | 2.38 ± 0.15 | | 2.40 ± 0.16 | | 2.27 ± 0.23 | 2.36 ± 0.16 | | 2.34 ± 0.11 | | 2.21 ± 0.35 | | 2.04 ± 0.34 | |
| **Rostral middle frontal** | | **2.25 ± 0.09** | | **2.32 ± 0.13** | | **2.16 ± 0.17^a, b^** | **2.24 ± 0.17** | | **2.22 ± 0.07** | | **2.06 ± 0.12 ^d^** | | **2.00 ± 0.23 ^d^** | |
| **Superior frontal** | | **2.56 ± 0.10** | | **2.68 ± 0.15** | | **2.43 ± 0.19^a, b^** | **2.61 ± 0.16** | | **2.44 ± 0.11 ^d^** | | **2.28 ± 0.15 ^d^** | | **2.24 ± 0.14 ^d^** | |
| Insula | | 2.91 ± 0.15 | | 2.92 ± 0.18 | | 2.85 ± 0.18 | 2.96 ± 0.15 | | 2.84 ± 0.09 | | 2.77 ± 0.20 | | 2.76 ± 0.31 | |
| Paracentral | | 2.43 ± 0.14 | | 2.39 ± 0.20 | | 2.29 ± 0.18 | 2.45 ± 0.18 | | 2.26 ± 0.05 | | 2.23 ± 0.22 | | 2.16 ± 0.22 | |
| **Precentral** | | **2.55 ± 0.16** | | **2.34± 0.19^c^** | | **2.41 ± 0.23^b^** | **2.60 ± 0.18** | | **2.38 ± 0.15** | | **2.41 ± 0.11** | | **2.20 ± 0.31** | |
| Cuneus | | 1.97 ± 0.11 | | 1.89 ± 0.16 | | 1.83 ± 0.17 | 1.94 ± 0.17 | | 1.77 ± 0.15 | | 1.89 ± 0.10 | | 1.76 ± 0.21 | |
| Lateral occipital | | 2.24 ± 0.13 | | 2.27 ± 0.14 | | 2.15 ± 0.14 | 2.25 ± 0.12 | | 2.14 ± 0.13 | | 2.12 ± 0.10 | | 2.03 ± 0.16 | |
| Pericalcarine | | 1.68 ± 0.18 | | 1.62 ± 0.11 | | 1.64 ± 0.22 | 1.77 ± 0.16 | | 1.58 ± 0.20 | | 1.71 ± 0.10 | | 1.58 ± 0.35 | |
| Inferior parietal | | 2.42 ± 0.13 | | 2.43 ± 0.14 | | 2.32 ± 0.18 | 2.47 ± 0.12 | | 2.32 ± 0.09 | | 2.27 ± 0.07 | | 2.13 ± 0.25 | |
| Postcentral | | 2.10 ± 0.14 | | 1.97 ± 0.16 | | 2.02 ± 0.21 | 2.12 ± 0.19 | | 2.06 ± 0.19 | | 1.99 ± 0.06 | | 1.80 ± 0.22 | |
| Precuneus | | 2.38 ± 0.11 | | 2.36 ± 0.17 | | 2.31 ± 0.19 | 2.45 ± 0.15 | | 2.34 ± 0.10 | | 2.17 ± 0.18 | | 2.12 ± 0.23 | |
| Superior parietal | | 2.19 ± 0.10 | | 2.20 ± 0.14 | | 2.10 ± 0.15 | 2.23 ± 0.11 | | 2.10 ± 0.10 | | 2.07 ± 0.10 | | 1.97 ± 0.19 | |
| Supramarginal | | 2.50 ± 0.11 | | 2.43 ± 0.17 | | 2.41 ± 0.22 | 2.58 ± 0.17 | | 2.42 ± 0.15 | | 2.37 ± 0.16 | | 2.20 ± 0.29 | |
| Banks superior temporal | | 2.54 ± 0.15 | | 2.51 ± 0.22 | | 2.40 ± 0.20 | 2.47 ± 0.24 | | 2.45 ± 0.16 | | 2.35 ± 0.27 | | 2.22 ± 0.12 | |
| Entorhinal | | 3.59 ± 0.38 | | 3.37± 0.52 | | 3.40 ± 0.36 | 3.39 ± 0.28 | | 3.55 ± 0.26 | | 3.35 ± 0.48 | | 3.13 ± 0.51 | |
| **Fusiform** | | **2.73 ± 0.15** | | **2.64± 0.14** | | **2.59 ± 0.20^b^** | **2.73 ± 0.21** | | **2.59 ± 0.15** | | **2.50 ± 0.17** | | **2.47 ± 0.22 ^d^** | |
| Inferior temporal | | 2.76 ± 0.16 | | 2.64± 0.11 | | 2.60 ± 0.18 | 2.77 ± 0.15 | | 2.62 ± 0.12 | | 2.38 ± 0.10 | | 2.45 ± 0.11 | |
| lingual | | 2.07 ± 0.13 | | 2.03± 0.17 | | 2.00 ± 0.16 | 2.10 ± 0.11 | | 1.97 ± 0.12 | | 1.99 ± 0.07 | | 1.91 ± 0.26 | |
| **Middle temporal** | | **2.80 ± 0.14** | | **2.88± 0.19** | | **2.73 ± 0.18^b^** | **2.88 ± 0.17** | | **2.72 ± 0.12** | | **2.64 ± 0.14** | | **2.58 ± 0.19 ^d^** | |
| Parahippocampal | | 2.73 ± 0.21 | | 2.60± 0.27 | | 2.57 ± 0.35 | 2.86 ± 0.35 | | 2.59 ± 0.26 | | 2.21 ± 0.15 | | 2.32 ± 0.27 | |
| Superior temporal | | 2.78± 0.16 | | 2.76± 0.22 | | 2.64 ± 0.21 | 2.78 ± 0.23 | | 2.65 ± 0.18 | | 2.53 ± 0.13 | | 2.48 ± 0.15 | |
| Temporal pole | | 3.75± 0.31 | | 3.69± 0.31 | | 3.62 ± 0.49 | 3.92 ± 0.43 | | 3.68 ± 0.44 | | 3.39 ± 0.34 | | 3.22 ± 0.53 | |
| Transverse temporal | | 2.52 ± 0.17 | | 2.36± 0.23 | | 2.45 ± 0.25 | 2.59 ± 0.28 | | 2.43 ± 0.27 | | 2.49 ± 0.17 | | 2.29 ± 0.09 | |
| **Left Hemisphere** | |  |  |  |  |  |  |  |  |  |  |  |  |  |
| Caudal anterior cingulate | | 2.59± 0.25 | | 2.57± 0.21 | | 2.52 ± 0.28 | 2.52 ± 0.31 | | 2.48 ± 0.30 | | 2.38 ± 0.30 | | 2.68 ± 0.13 | |
| Isthmus cingulate | | 2.48± 0.19 | | 2.43± 0.20 | | 2.36 ± 0.21 | 2.50 ± 0.12 | | 2.38 ± 0.20 | | 2.28 ± 0.18 | | 2.15 ± 0.17 | |
| Posterior cingulate | | 2.49± 0.14 | | 2.53± 0.11 | | 2.43 ± 0.19 | 2.41 ± 0.13 | | 2.50 ± 0.13 | | 2.20 ± 0.24 | | 2.44 ± 0.28 | |
| Rostral anterior cingulate | | 2.71± 0.22 | | 2.77± 0.24 | | 2.71 ± 0.23 | 2.80 ± 0.15 | | 2.68 ± 0.27 | | 2.58 ± 0.20 | | 2.72 ± 0.26 | |
| **Caudal middle frontal** | | **2.47± 0.09** | | **2.49± 0.12** | | **2.35 ± 0.19^a, b^** | **2.49 ± 0.20** | | **2.39 ± 0.12** | | **2.23 ± 0.14 ^d^** | | **2.14 ± 0.15 ^d^** | |
| Frontal pole | | 2.73± 0.21 | | 2.71± 0.28 | | 2.57 ± 0.30 | 2.72 ± 0.31 | | 2.64 ± 0.26 | | 2.30 ± 0.31 | | 2.35 ± 0.19 | |
| **Lateral orbitofrontal** | | **2.45± 0.12** | | **2.60± 0.12** | | **2.39 ± 0.20^b^** | **2.55 ± 0.19** | | **2.42 ± 0.13 ^d^** | | **2.24 ± 0.10 ^d^** | | **2.19 ± 0.18 ^d^** | |
| Medial orbitofrontal | | 2.37± 0.12 | | 2.35± 0.19 | | 2.36 ± 0.21 | 2.44 ± 0.16 | | 2.37 ± 0.14 | | 2.25 ± 0.10 | | 2.26 ± 0.39 | |
| Pars opercularis | | 2.49± 0.11 | | 2.49± 0.15 | | 2.40 ± 0.19 | 2.52 ± 0.20 | | 2.40 ± 0.14 | | 2.36 ± 0.19 | | 2.26 ± 0.20 | |
| Pars orbitalis | | 2.66± 0.15 | | 2.68± 0.18 | | 2.51 ± 0.25 | 2.69 ± 0.23 | | 2.57 ± 0.11 | | 2.34 ± 0.28 | | 2.22 ± 0.19 | |
| Pars triangularis | | 2.37± 0.12 | | 2.36± 0.12 | | 2.28 ± 0.24 | 2.42 ± 0.27 | | 2.31 ± 0.14 | | 2.18 ± 0.21 | | 2.04 0.25 | |
| **Rostral middle frontal** | | **2.30± 0.09** | | **2.32± 0.10** | | **2.18 ± 0.18^a, b^** | **2.29 ± 0.17** | | **2.22 ± 0.11** | | **2.09 ± 0.13 ^d^** | | **2.00 ± 0.23 ^d^** | |
| **Superior frontal** | | **2.59± 0.09** | | **2.70± 0.13** | | **2.47 ± 0.24^b^** | **2.68 ± 0.20** | | **2.50 ± 0.13 ^d^** | | **2.29 ± 0.18 ^d^** | | **2.23 ± 0.22 ^d^** | |
| Insula |  | 2.95± 0.16 | | 2.94 ± 0.16 | | 2.89 ± 0.21 | 3.01 ± 0.18 | | 2.90 ± 0.15 | | 2.79 ± 0.26 | | 2.76 ± 0.31 | |
| Paracentral | | 2.44 ± 0.14 | | 2.37 ± 0.23 | | 2.32 ± 0.18 | 2.50 ± 0.14 | | 2.32 ± 0.06 | | 2.27 ± 0.19 | | 2.13 ± 0.20 | |
| Precentral | | 2.56± 0.17 | | 2.40± 0.16 | | 2.45 ± 0.22 | 2.64 ± 0.19 | | 2.42 ± 0.14 | | 2.44 ± 0.11 | | 2.23 ± 0.22 | |
| Cuneus |  | 1.92± 0.13 | | 1.86 ± 0.14 | | 1.84 ± 0.13 | 1.90 ± 0.14 | | 1.81 ± 0.12 | | 1.88 ± 0.03 | | 1.79 ± 0.18 | |
| **Lateral occipital** | | **2.20± 0.11** | | **2.20± 0.15** | | **2.08 ± 0.13^a, b^** | **2.14 ± 0.17** | | **2.07 ± 0.12** | | **2.09 ± 0.05** | | **2.00 ± 0.13 ^d^** | |
| Pericalcarine | | 1.74± 0.15 | | 1.63± 0.17 | | 1.64 ± 0.17 | 1.70 ± 0.18 | | 1.63 ± 0.14 | | 1.67 ± 0.10 | | 1.58 ± 0.26 | |
| **Inferior parietal** | | **2.42± 0.10** | | **2.45± 0.16** | | **2.30 ± 0.16^a, b^** | **2.44 ± 0.14** | | **2.30 ± 0.10 ^d^** | | **2.23 ± 0.05** | | **2.15 ± 0.22 ^d^** | |
| Postcentral | | 2.12± 0.12 | | 1.99± 0.13 | | 2.06 ± 0.15 | 2.18 ± 0.12 | | 2.07 ± 0.12 | | 2.03 ± 0.04 | | 1.90 ± 0.18 | |
| Precuneus | | 2.35± 0.10 | | 2.35± 0.12 | | 2.26 ± 0.17 | 2.41 ± 0.15 | | 2.26 ± 0.15 | | 2.13 ± 0.14 | | 2.11 ± 0.11 | |
| **Superior parietal** | | **2.19± 0.07** | | **2.21± 0.14** | | **2.09 ± 0.13^a, b^** | **2.20 ± 0.12** | | **2.09 ± 0.09** | | **2.07 ± 0.09** | | **1.96 ± 0.14 ^d^** | |
| Supramarginal | | 2.51± 0.12 | | 2.51± 0.14 | | 2.38 ± 0.20 | 2.54 ± 0.14 | | 2.39 ± 0.08 | | 2.34 ± 0.14 | | 2.17 ± 0.28 | |
| Banks superior temporal |  | 2.45± 0.15 | | 2.48± 0.22 | | 2.34 ± 0.16 | 2.42 ± 0.18 | | 2.36 ± 0.15 | | 2.24 ± 0.14 | | 2.26 ± 0.10 | |
| Entorhinal | | 3.51± 0.40 | | 3.32± 0.37 | | 3.24 ± 0.34 | 3.47 ± 0.25 | | 3.33 ± 0.26 | | 2.98 ± 0.48 | | 2.90 ± 0.22 | |
| Fusiform |  | 2.74± 0.14 | | 2.65± 0.15 | | 2.63 ± 0.20 | 2.79 ± 0.20 | | 2.64 ± 0.11 | | 2.49 ± 0.13 | | 2.46 ± 0.23 | |
| Inferior temporal | | 2.77± 0.15 | | 2.60± 0.16 | | 2.62 ± 0.22 | 2.79 ± 0.20 | | 2.63 ± 0.17 | | 2.44 ± 0.20 | | 2.46 ± 0.22 | |
| Lingual |  | 2.00± 0.18 | | 2.02± 0.14 | | 1.93 ± 0.14 | 1.95 ± 0.15 | | 1.94 ± 0.13 | | 1.93 ± 0.01 | | 1.85 ± 0.20 | |
| Middle temporal | | 2.84± 0.16 | | 2.84± 0.20 | | 2.74 ± 0.21 | 2.90 ± 0.21 | | 2.78 ± 0.15 | | 2.61 ± 0.10 | | 2.52 ± 0.13 | |
| Parahippocampal | | 2.76 0.30 | | 2.67± 0.24 | | 2.65 ± 0.38 | 3.02 ± 0.26 | | 2.59 ± 0.35 | | 2.39 ± 0.31 | | 2.43 ± 0.29 | |
| Superior temporal | | 2.76± 0.18 | | 2.77± 0.20 | | 2.64 ± 0.21 | 2.78 ± 0.26 | | 2.65 ± 0.16 | | 2.59 ± 0.14 | | 2.48 ± 0.15 | |
| Temporal pole | | 3.67± 0.26 | | 3.65± 0.34 | | 3.42 ± 0.45 | 3.71 ± 0.51 | | 3.44 ± 0.39 | | 3.07 ± 0.40 | | 3.17 ± 0.36 | |
| Transverse temporal | | 2.45± 0.18 | | 2.29± 0.23 | | 2.43 ± 0.21 | 2.51 ± 0.34 | | 2.39 ± 0.16 | | 2.41 ± 0.17 | | 2.44 ± 0.13 | |

Mean± SD. Healthy controls (HC) and sALS and C9 group+ as a whole were first compared, with age and gender as covariates. Regions in BOLD differed between groups, p < 0.05 corrected for multiple comparisons. **^a^**C9+ group < HC; **^b^**C9+ < sALS; **^c^**sALS < HC;

Dunnett’s post-hoc testing was carried out on significant regions (in BOLD) to identify differences between C9+ subgroups and sALS patients.

**^d^**C9+ subgroup < sALS; p < 0.05
