# Supplementary material for: Longitudinal imaging in C9orf72 mutation carriers: Relationship to phenotype
Source: Neuroimage Clin. 2016 Oct 22;12:1035–43. doi: 10.1016/j.nicl.2016.10.014 (PMC5153604; doi:10.1016/j.nicl.2016.10.014)
Supplement: Supplemental Table 2 — Age-adjusted correlations of ALSFRS-R and Letter Fluency scores with regional cortical thickness. [file mmc2.docx]

Suppl Table 2. Age adjusted correlations of ALSFRS-R and Verbal Fluency scores with regional cortical thickness

| Cortical Region | ALSFRS-R  r value  (N=45) | p value | Fluency  r value  (N=37) | p value |
| --- | --- | --- | --- | --- |
| **Right Hemisphere** |  |  |  |  |
| caudal anterior cingulate | 0.037 | 0.805 | -0.137 | 0.406 |
| isthmus cingulate | 0.001 | 0.996 | 0.07 | 0.671 |
| posterior cingulate | -0.019 | 0.899 | -0.004 | 0.982 |
| rostral anterior cingulate | -0.14 | 0.347 | -0.042 | 0.8 |
| caudal middle frontal | -0.041 | 0.784 | **0.508** | **0.001** |
| frontal pole | -0.145 | 0.329 | 0.111 | 0.499 |
| lateral orbitofrontal | -0.058 | 0.698 | 0.299 | 0.065 |
| medial orbitofrontal | -0.108 | 0.469 | 0.247 | 0.129 |
| pars opercularis | -0.012 | 0.937 | **0.571** | **<0.001** |
| par sorbitalis | -0.057 | 0.702 | **0.656** | **<0.001** |
| pars triangularis | -0.037 | 0.808 | 0.373 | 0.019 |
| rostral middle frontal | -0.085 | 0.568 | 0.449 | 0.004 |
| superior frontal | -0.15 | 0.313 | 0.460 | 0.003 |
| insula | -0.072 | 0.629 | 0.273 | 0.092 |
| paracentral | 0.133 | 0.373 | 0.394 | 0.013 |
| precentral | 0.389 | 0.007 | 0.341 | 0.033 |
| cuneus | 0.182 | 0.222 | 0.367 | 0.021 |
| lateral occipital | -0.008 | 0.957 | 0.297 | 0.066 |
| pericalcarine | 0.131 | 0.382 | 0.158 | 0.336 |
| inferior parietal | -0.028 | 0.851 | 0.374 | 0.019 |
| postcentral | 0.083 | 0.58 | 0.393 | 0.013 |
| precuneus | -0.018 | 0.905 | 0.335 | 0.037 |
| superior parietal | -0.038 | 0.802 | 0.379 | 0.017 |
| supramarginal | 0.115 | 0.442 | 0.384 | 0.016 |
| banks sup. temp | -0.156 | 0.295 | 0.208 | 0.204 |
| entorhinal | -0.106 | 0.477 | 0.115 | 0.486 |
| fusiform | 0.076 | 0.61 | 0.341 | 0.034 |
| inferior temporal | -0.078 | 0.604 | 0.325 | 0.043 |
| lingual | 0.099 | 0.507 | 0.287 | 0.077 |
| middle temporal | -0.134 | 0.367 | 0.258 | 0.113 |
| parahippocampal | 0.153 | 0.305 | 0.330 | 0.040 |
| superior temporal | -0.046 | 0.758 | 0.304 | 0.06 |
| Temporal pole | 0.136 | 0.361 | 0.302 | 0.062 |
| transverse temporal | 0.208 | 0.161 | 0.329 | 0.041 |
| **Left Hemisphere** |  |  |  |  |
| caudal anterior cingulate | -0.041 | 0.784 | -0.159 | 0.333 |
| isthmus cingulate | 0.001 | 0.997 | 0.035 | 0.832 |
| posterior cingulate | -0.252 | 0.087 | 0.222 | 0.175 |
| rostral anterior cingulate | -0.055 | 0.714 | 0.043 | 0.794 |
| caudal middle frontal | -0.053 | 0.723 | **0.527** | **0.001** |
| frontal pole | -0.137 | 0.358 | 0.285 | 0.079 |
| lateral orbitofrontal | -0.169 | 0.256 | 0.413 | 0.009 |
| medial orbitofrontal | 0.151 | 0.31 | 0.026 | 0.875 |
| pars opercularis | 0.036 | 0.812 | **0.519** | **0.001** |
| par sorbitalis | -0.005 | 0.975 | 0.373 | 0.019 |
| pars triangularis | -0.017 | 0.909 | 0.388 | 0.015 |
| rostral middle frontal | -0.093 | 0.535 | 0.36 | 0.024 |
| superior frontal | -0.122 | 0.414 | 0.494 | 0.001 |
| insula | 0.015 | 0.92 | 0.264 | 0.104 |
| paracentral | 0.263 | 0.074 | 0.478 | 0.002 |
| precentral | 0.394 | 0.006 | 0.327 | 0.042 |
| cuneus | 0.105 | 0.484 | 0.195 | 0.235 |
| lateral occipital | 0.02 | 0.895 | 0.299 | 0.065 |
| pericalcarine | 0.13 | 0.383 | 0.198 | 0.227 |
| inferior parietal | -0.009 | 0.953 | 0.400 | 0.012 |
| postcentral | 0.217 | 0.143 | 0.441 | 0.005 |
| precuneus | -0.11 | 0.462 | 0.413 | 0.009 |
| superior parietal | -0.081 | 0.587 | 0.415 | 0.009 |
| supramarginal | 0.031 | 0.838 | 0.398 | 0.012 |
| banks sup. temp | -0.052 | 0.729 | 0.337 | 0.036 |
| entorhinal | -0.082 | 0.582 | **0.508** | **0.001** |
| fusiform | 0.134 | 0.371 | 0.292 | 0.071 |
| inferior temporal | 0.207 | 0.162 | 0.401 | 0.012 |
| lingual | -0.055 | 0.712 | 0.292 | 0.072 |
| middle temporal | -0.145 | 0.331 | 0.414 | 0.009 |
| parahippocampal | 0.341 | 0.019 | 0.319 | 0.048 |
| superior temporal | -0.008 | 0.956 | 0.329 | 0.041 |
| Temporal pole | 0.091 | 0.545 | 0.354 | 0.027 |
| transverse temporal | 0.309 | 0.035 | 0.057 | 0.729 |
