# Supplementary material for: Longitudinal imaging in C9orf72 mutation carriers: Relationship to phenotype
Source: Neuroimage Clin. 2016 Oct 22;12:1035–43. doi: 10.1016/j.nicl.2016.10.014 (PMC5153604; doi:10.1016/j.nicl.2016.10.014)
Supplement: Supplemental Table 3 — Cortical regions with highly reproducible scan-rescan thickness measures (r > 0.90) in healthy controls. [file mmc3.docx]

Supplemental Table 3. Cortical regions with highly reproducible thickness measures (r > 0.90) on repeat scans of healthy controls

| Left Hemisphere | r |  | Right Hemisphere | r |
| --- | --- | --- | --- | --- |
| Precentral | .905^**^ |  | Precentral | .904^**^ |
| Insula | .929^**^ |  | Insula | .907^**^ |
| Pars orbitalis | .921^**^ |  | Pars orbitalis | .943^**^ |
| Lateral orbitofrontal | .914^**^ |  |  |  |
| Medial orbitofrontal | .920^**^ |  | Pars triangularis | .961^**^ |
| Frontal pole | .940^**^ |  | Pars opercularis | .913^**^ |
|  |  |  |  |  |
| Banks superior temporal | .935^**^ |  | Banks superior temporal | .949^**^ |
| Entorhinal | .912^**^ |  | Entorhinal | .915^**^ |
| Inferior temporal | .920^**^ |  | Inferior temporal | .930^**^ |
| Transverse temporal | .915^**^ |  | Transverse temporal | .923^**^ |
| Superior temporal | .964^**^ |  | Superior temporal | .952^**^ |
| Middle temporal | .929^**^ |  |  |  |
| Lingual | .919^**^ |  |  |  |
| Temporal pole | .920^**^ |  |  |  |
| Parahippocampal | .959^**^ |  |  |  |
|  |  |  |  |  |
| Posterior cingulate | .903^**^ |  | Posterior cingulate | .945^**^ |
| Isthmus cingulate | .905^**^ |  | Isthmus cingulate | .945^**^ |
| Supramarginal | .966^**^ |  | Inferior parietal | .916^**^ |
|  |  |  |  |  |
|  |  |  | Lateral occipital | .905^**^ |

***p < 0.001
